# Methyl 2-(2-methyl-4-nitro-1*H*-imidazol-1-yl)acetate

**DOI:** 10.1107/S1600536813011914

**Published:** 2013-05-04

**Authors:** Sana Zama, Abdelmalek Bouraiou, Sofiane Bouacida, Thierry Roisnel, Ali Belfaitah

**Affiliations:** a Laboratoire des Produits Naturels d’Origine Végétale et de Synthèse Organique, PHYSYNOR, Université Constantine, 25000 Constantine, Algeria; b Unité de Recherche de Chimie de l’Environnement et Moléculaire Structurale, CHEMS, Université Constantine, 25000 Constantine, Algeria; c Département des Sciences de la Matière, Faculté des Sciences Exactes et Sciences de la Nature et de la Vie, Université Oum El Bouaghi, 04000 Oum El Bouaghi, Algeria; d Centre de Diffractométrie X, UMR 6226 CNRS, Unité Sciences Chimiques de Rennes, Université de Rennes I, 263 Avenue du Général Leclerc, 35042 Rennes, France

## Abstract

In the crystal of the title compound, C_7_H_9_N_3_O_4_, mol­ecules are linked by weak C—H⋯O hydrogen bonds into chains along the *a*-axis direction. The dihedral angle between the ring and the nitro group is 3.03 (6), while that between the ring and the acetate group is 85.01 (3)°.

## Related literature

For the synthesis of the title compound, see: Pavlik *et al.* (2011 ▶); Kasai *et al.* (2001 ▶). For the structural identification of nitro­imidazoles, see: Larina & Lopyrev (2009 ▶). For biological activities of this class of compounds, see: Gaonkar *et al.* (2009 ▶); Olender *et al.* (2009 ▶). For our previous work on imidazole derivatives, see: Chelghoum *et al.* (2011 ▶); Bahnous *et al.* (2012 ▶).
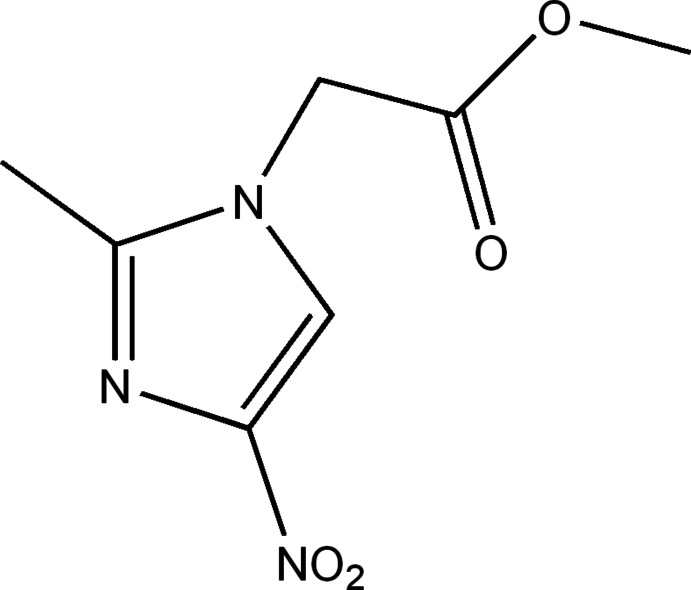


## Experimental

### 

#### Crystal data

C_7_H_9_N_3_O_4_
*M* *_r_* = 199.17
Monoclinic, 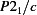

*a* = 4.6619 (2) Å
*b* = 17.3256 (7) Å
*c* = 11.1490 (4) Å
β = 103.204 (2)°
*V* = 876.70 (6) Å^3^
*Z* = 4
Mo *K*α radiation
μ = 0.13 mm^−1^
*T* = 150 K
0.35 × 0.3 × 0.12 mm

#### Data collection

Bruker APEXII CCD diffractometer
Absorption correction: multi-scan (*SADABS*; Bruker, 2002 ▶) *T*_min_ = 0.937, *T*_max_ = 0.985
7665 measured reflections
1991 independent reflections
1753 reflections with *I* > 2σ(*I*)
*R*_int_ = 0.030

#### Refinement

*R*[*F* ^2^ > 2σ(*F* ^2^)] = 0.034
*wR*(*F* ^2^) = 0.088
*S* = 1.09
1991 reflections
129 parameters
H-atom parameters constrained
Δρ_max_ = 0.26 e Å^−3^
Δρ_min_ = −0.21 e Å^−3^

### 

Data collection: *APEX2* (Bruker, 2004 ▶); cell refinement: *SAINT* (Bruker, 2004 ▶); data reduction: *SAINT*; program(s) used to solve structure: *SIR2002* (Burla *et al.*, 2005 ▶); program(s) used to refine structure: *SHELXL97* (Sheldrick, 2008 ▶); molecular graphics: *ORTEP-3 for Windows* (Farrugia, 2012 ▶) and *DIAMOND* (Brandenburg & Berndt, 2001 ▶); software used to prepare material for publication: *WinGX* (Farrugia, 2012 ▶) and *CRYSCAL* (local program).

## Supplementary Material

Crystal structure: contains datablock(s) global, I. DOI: 10.1107/S1600536813011914/yk2093sup1.cif

Structure factors: contains datablock(s) I. DOI: 10.1107/S1600536813011914/yk2093Isup2.hkl

Supplementary material file. DOI: 10.1107/S1600536813011914/yk2093Isup3.cml

Additional supplementary materials:  crystallographic information; 3D view; checkCIF report

## Figures and Tables

**Table 1:** Hydrogen-bond geometry (Å, °)

*D*—H⋯*A*	*D*—H	H⋯*A*	*D*⋯*A*	*D*—H⋯*A*
C5—H5*A*⋯O4^i^	0.99	2.30	3.2350 (16)	156

Symmetry code: (i) 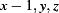.

**Fig. 1.**
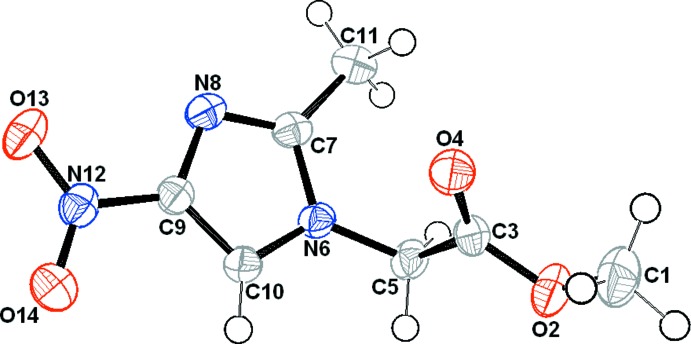
A view of the title molecule with the atom-labelling scheme. Displacement ellipsoids are drawn at the 50% probability level. H atoms are represented as small spheres of arbitrary radius.

**Fig. 2.**
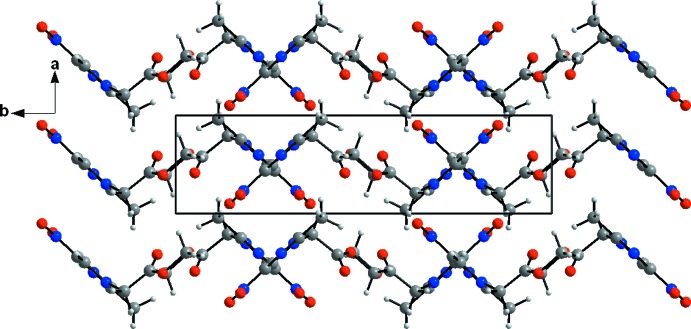
The crossed layers in the structure of the title compound, viewed down the c axis.

**Fig. 3.**
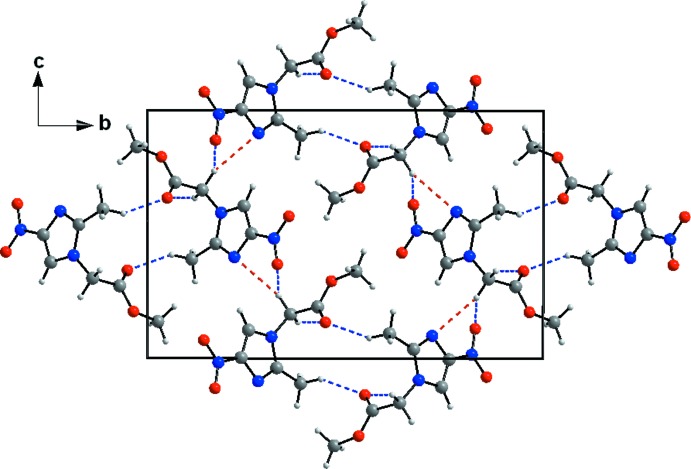
A diagram of the layered crystal packing of the title compound, viewed down the a axis and showing hydrogen bonds as dashed lines.

## supplementary crystallographic information

### Comment

The chemistry of imidazole occupies an extremely important position within the family of five-membered heterocyclic compounds. Synthesis of imidazole derivatives has attracted great interest in recent years due to their broad spectrum of biological activities (Gaonkar *et al.*, 2009). For a few decades, nitroimdazoles have been the subject of much interest because of their properties. Nitroimidazoles, such as metronidazole, misonidazole, ornidazole, secnidazole and etamidazole, are commonly used as therapeutic agents against a variety of protozoan and bacterial infections of humans and animals (Olender *et al.*, 2009). The compounds with nitro group at position 4 are usually less active than the corresponding 5-nitro derivatives. The structures of nitroimidazoles have been studied more thoroughly by X-ray and ^13^C NMR and can be explained by wide application of these compounds, especially in medicine. The problem of structural identification of 1-substituted nitroimidazoles by NMR spectroscopy has been considered in many works (Larina & Lopyrev, 2009). In previous work, we have reported the synthesis and structure determination of some new heterocyclic compounds bearing an imidazole unit (Chelghoum *et al.*, 2011; Bahnous *et al.*, 2012). Herein, we report the synthesis and single-crystal X-ray structure of methyl 2-(2-methyl-4-nitro-1*H*-imidazol-1-yl)acetate, (I).

The molecular geometry and the atom-numbering scheme of (I) are shown in Fig. 1. The asymmetric unit of (I)consists of 2-methyl-4-nitro-1*H*-imidazol linked to methyl acetate. The crystal packing can be described as crossed layers in zigzag parallel to the (110) plane. (Fig. 2). It is stabilized by C—H···O and C—H···N hydrogen bond (Table 1), weak N—O···π interactions and π–π stacking interactions between imidazole rings with a centroid–centroid distance of 4.6619 (6)Å. These interaction bonds link the molecules within the layers and also link the layers together, reinforcing the cohesion of the structure.

### Experimental

Methyl 2-(2-methyl-4-nitro-1*H*-imidazol-1-yl)acetate, (I), was obtained from reaction of methyl bromoacetate and 4(5)-nitro-2-methylimidazole in presence of potassium carbonate in DMF. Crystals suitable for X-ray analysis were obtained by slow evaporation of a chloroform–carbon tetrachloride solution.

### Refinement

All non-H atoms were refined with anisotropic atomic displacement parameters. Approximate positions for all the H atoms were first obtained from the difference electron-density map. However, the H atoms were situated into idealized positions and the H atoms have been refined within the riding-atom approximation. The applied constraints were as follows: C_aryl_—H_aryl_ = 0.95 Å, C_methylene_—H_methylene_ = 0.99 Å and C_methyl_—H_methyl_ = 0.98 Å. The idealized methyl group was allowed to rotate about the C—C bond during the refinement by application of the command AFIX 137 in *SHELXL97* (Sheldrick, 2008). *U*_iso_(H_methyl_) = 1.5*U*_eq_(C_methyl_) or *U*_iso_(H_aryl_ or H_methylene_) = 1.2*U*_eq_(C_aryl_ or C_methylene_).

### Crystal data

**Table:**

C_7_H_9_N_3_O_4_	*F*(000) = 416
*M**_r_* = 199.17	*D*_x_ = 1.509 Mg m^−^^3^
Monoclinic, *P*2_1_/*c*	Mo *K*α radiation, λ = 0.71073 Å
Hall symbol: -P 2ybc	Cell parameters from 3719 reflections
*a* = 4.6619 (2) Å	θ = 3.8–27.5°
*b* = 17.3256 (7) Å	µ = 0.13 mm^−^^1^
*c* = 11.1490 (4) Å	*T* = 150 K
β = 103.204 (2)°	Prism, colourless
*V* = 876.70 (6) Å^3^	0.35 × 0.3 × 0.12 mm
*Z* = 4	

### Data collection

**Table:**

Bruker APEXII CCD diffractometer	1753 reflections with *I* > 2σ(*I*)
Graphite monochromator	*R*_int_ = 0.030
CCD rotation images, thin slices scans	θ_max_ = 27.5°, θ_min_ = 3.8°
Absorption correction: multi-scan (*SADABS*; Bruker, 2002)	*h* = −6→5
*T*_min_ = 0.937, *T*_max_ = 0.985	*k* = −22→22
7665 measured reflections	*l* = −14→13
1991 independent reflections	

### Refinement

**Table:**

Refinement on *F*^2^	Primary atom site location: structure-invariant direct methods
Least-squares matrix: full	Secondary atom site location: difference Fourier map
*R*[*F*^2^ > 2σ(*F*^2^)] = 0.034	Hydrogen site location: inferred from neighbouring sites
*wR*(*F*^2^) = 0.088	H-atom parameters constrained
*S* = 1.09	*w* = 1/[σ^2^(*F*_o_^2^) + (0.0409*P*)^2^ + 0.2051*P*] where *P* = (*F*_o_^2^ + 2*F*_c_^2^)/3
1991 reflections	(Δ/σ)_max_ = 0.002
129 parameters	Δρ_max_ = 0.26 e Å^−^^3^
0 restraints	Δρ_min_ = −0.21 e Å^−^^3^

### Special details

**Table:**

Geometry. All s.u.'s (except the s.u. in the dihedral angle between two l.s. planes) are estimated using the full covariance matrix. The cell s.u.'s are taken into account individually in the estimation of s.u.'s in distances, angles and torsion angles; correlations between s.u.'s in cell parameters are only used when they are defined by crystal symmetry. An approximate (isotropic) treatment of cell s.u.'s is used for estimating s.u.'s involving l.s. planes.
Refinement. Refinement of *F*^2^ against ALL reflections. The weighted *R*-factor *wR* and goodness of fit *S* are based on *F*^2^, conventional *R*-factors *R* are based on *F*, with *F* set to zero for negative *F*^2^. The threshold expression of *F*^2^ > σ(*F*^2^) is used only for calculating *R*-factors(gt) *etc*. and is not relevant to the choice of reflections for refinement. *R*-factors based on *F*^2^ are statistically about twice as large as those based on *F*, and *R*-factors based on ALL data will be even larger.

### Fractional atomic coordinates and isotropic or equivalent isotropic displacement parameters (Å^2^)

**Table:**

	*x*	*y*	*z*	*U*_iso_*/*U*_eq_	
C1	0.6041 (3)	−0.02718 (8)	0.85178 (13)	0.0358 (3)	
H1A	0.5668	−0.0689	0.7909	0.054*	
H1B	0.5828	−0.0469	0.9317	0.054*	
H1C	0.8046	−0.0075	0.8596	0.054*	
O2	0.39315 (19)	0.03498 (5)	0.81191 (8)	0.0303 (2)	
C3	0.4191 (2)	0.06977 (6)	0.70857 (10)	0.0215 (2)	
O4	0.58536 (18)	0.05011 (5)	0.64611 (8)	0.0268 (2)	
C5	0.2106 (3)	0.13799 (6)	0.67831 (10)	0.0227 (2)	
H5A	0.0069	0.1194	0.6459	0.027*	
H5B	0.2144	0.1684	0.7538	0.027*	
N6	0.3015 (2)	0.18625 (5)	0.58664 (8)	0.0199 (2)	
C7	0.2472 (2)	0.17282 (6)	0.46184 (10)	0.0201 (2)	
N8	0.4024 (2)	0.21960 (5)	0.40814 (8)	0.0215 (2)	
C9	0.5615 (2)	0.26266 (6)	0.50325 (10)	0.0199 (2)	
C10	0.5056 (2)	0.24382 (6)	0.61419 (10)	0.0211 (2)	
H10	0.5892	0.2657	0.6925	0.025*	
C11	0.0408 (3)	0.11215 (7)	0.39924 (11)	0.0277 (3)	
H11A	−0.0054	0.1211	0.3101	0.042*	
H11B	−0.1411	0.1141	0.4292	0.042*	
H11C	0.1327	0.0613	0.4172	0.042*	
N12	0.7683 (2)	0.31975 (5)	0.48349 (9)	0.0228 (2)	
O13	0.80836 (19)	0.32840 (5)	0.37915 (8)	0.0315 (2)	
O14	0.8988 (2)	0.35710 (5)	0.57330 (8)	0.0361 (2)	

### Atomic displacement parameters (Å^2^)

**Table:**

	*U*^11^	*U*^22^	*U*^33^	*U*^12^	*U*^13^	*U*^23^
C1	0.0406 (7)	0.0303 (7)	0.0373 (7)	0.0065 (6)	0.0110 (6)	0.0126 (6)
O2	0.0335 (5)	0.0293 (5)	0.0315 (5)	0.0039 (4)	0.0143 (4)	0.0106 (4)
C3	0.0218 (5)	0.0203 (5)	0.0228 (5)	−0.0043 (4)	0.0061 (4)	0.0001 (4)
O4	0.0290 (4)	0.0248 (4)	0.0293 (4)	0.0039 (3)	0.0124 (4)	0.0003 (3)
C5	0.0231 (6)	0.0243 (6)	0.0235 (6)	0.0000 (4)	0.0112 (4)	0.0016 (4)
N6	0.0223 (5)	0.0196 (4)	0.0191 (5)	0.0021 (4)	0.0075 (4)	−0.0002 (3)
C7	0.0198 (5)	0.0208 (5)	0.0197 (5)	0.0062 (4)	0.0045 (4)	−0.0001 (4)
N8	0.0232 (5)	0.0230 (5)	0.0185 (5)	0.0051 (4)	0.0054 (4)	0.0013 (4)
C9	0.0215 (5)	0.0177 (5)	0.0215 (5)	0.0037 (4)	0.0068 (4)	0.0014 (4)
C10	0.0243 (5)	0.0198 (5)	0.0200 (5)	0.0003 (4)	0.0067 (4)	−0.0009 (4)
C11	0.0265 (6)	0.0282 (6)	0.0276 (6)	0.0002 (5)	0.0043 (5)	−0.0051 (5)
N12	0.0238 (5)	0.0212 (5)	0.0245 (5)	0.0040 (4)	0.0080 (4)	0.0037 (4)
O13	0.0340 (5)	0.0365 (5)	0.0279 (5)	−0.0001 (4)	0.0154 (4)	0.0069 (4)
O14	0.0417 (6)	0.0349 (5)	0.0318 (5)	−0.0141 (4)	0.0084 (4)	−0.0049 (4)

### Geometric parameters (Å, º)

**Table:**

C1—O2	1.4578 (15)	C7—N8	1.3178 (15)
C1—H1A	0.98	C7—C11	1.4869 (16)
C1—H1B	0.98	N8—C9	1.3682 (15)
C1—H1C	0.98	C9—C10	1.3607 (15)
O2—C3	1.3299 (13)	C9—N12	1.4329 (14)
C3—O4	1.2036 (13)	C10—H10	0.95
C3—C5	1.5183 (16)	C11—H11A	0.98
C5—N6	1.4564 (14)	C11—H11B	0.98
C5—H5A	0.99	C11—H11C	0.98
C5—H5B	0.99	N12—O13	1.2287 (12)
N6—C10	1.3641 (15)	N12—O14	1.2302 (13)
N6—C7	1.3760 (14)		
			
O2—C1—H1A	109.5	N8—C7—N6	111.22 (10)
O2—C1—H1B	109.5	N8—C7—C11	125.81 (10)
H1A—C1—H1B	109.5	N6—C7—C11	122.96 (10)
O2—C1—H1C	109.5	C7—N8—C9	103.89 (9)
H1A—C1—H1C	109.5	C10—C9—N8	113.02 (10)
H1B—C1—H1C	109.5	C10—C9—N12	125.50 (10)
C3—O2—C1	114.24 (9)	N8—C9—N12	121.46 (10)
O4—C3—O2	124.82 (11)	C9—C10—N6	103.85 (10)
O4—C3—C5	123.76 (10)	C9—C10—H10	128.1
O2—C3—C5	111.41 (9)	N6—C10—H10	128.1
N6—C5—C3	109.20 (9)	C7—C11—H11A	109.5
N6—C5—H5A	109.8	C7—C11—H11B	109.5
C3—C5—H5A	109.8	H11A—C11—H11B	109.5
N6—C5—H5B	109.8	C7—C11—H11C	109.5
C3—C5—H5B	109.8	H11A—C11—H11C	109.5
H5A—C5—H5B	108.3	H11B—C11—H11C	109.5
C10—N6—C7	108.01 (9)	O13—N12—O14	123.54 (10)
C10—N6—C5	124.16 (9)	O13—N12—C9	118.87 (10)
C7—N6—C5	126.63 (10)	O14—N12—C9	117.58 (9)
			
C1—O2—C3—O4	4.91 (17)	C11—C7—N8—C9	−178.43 (10)
C1—O2—C3—C5	−175.23 (10)	C7—N8—C9—C10	−0.55 (12)
O4—C3—C5—N6	−15.79 (15)	C7—N8—C9—N12	178.05 (9)
O2—C3—C5—N6	164.35 (9)	N8—C9—C10—N6	−0.03 (12)
C3—C5—N6—C10	−86.81 (12)	N12—C9—C10—N6	−178.57 (10)
C3—C5—N6—C7	79.17 (13)	C7—N6—C10—C9	0.58 (12)
C10—N6—C7—N8	−0.99 (12)	C5—N6—C10—C9	168.78 (10)
C5—N6—C7—N8	−168.81 (10)	C10—C9—N12—O13	176.38 (10)
C10—N6—C7—C11	178.38 (10)	N8—C9—N12—O13	−2.04 (15)
C5—N6—C7—C11	10.55 (16)	C10—C9—N12—O14	−2.92 (16)
N6—C7—N8—C9	0.92 (11)	N8—C9—N12—O14	178.66 (10)

### Hydrogen-bond geometry (Å, º)

**Table:**

*D*—H···*A*	*D*—H	H···*A*	*D*···*A*	*D*—H···*A*
C5—H5*A*···O4^i^	0.99	2.30	3.2350 (16)	156

Symmetry code: (i) *x*−1, *y*, *z*.
